# Public health concern of antimicrobial resistance and virulence determinants in *E. coli* isolates from oysters in Egypt

**DOI:** 10.1038/s41598-024-77519-y

**Published:** 2024-11-06

**Authors:** Rahma Mohammed, Sara M. Nader, Dalia A. Hamza, Maha A. Sabry

**Affiliations:** Department of Zoonoses, Faculty of Veterinary Medicine, Cairo University, PO Box 12211, Giza, Egypt

**Keywords:** Multi-drug resistance, Extended-spectrum β-lactamases, Plasmid AmpC, Carbapenems, Virulence factors, *E. Coli* pathotypes, Antimicrobials, Bacteria

## Abstract

The emergence of critical-priority *E. coli*, carrying a wide array of resistance and virulence factors through food sources, poses a significant challenge to public health. This study aimed to investigate the potential role of oysters sold in Egypt as a source for *E. coli*, identify their resistance and virulence-associated gene profiles, and assess associated zoonotic risks. A total of 33 pooled fresh oyster samples were obtained from various retail fish markets in Egypt and examined bacteriologically for the presence of *E. coli*. Antimicrobial resistance was performed by the disk-diffusion method, and the multiple antibiotic resistance index (MAR) was calculated. All isolates were screened for extended-spectrum beta-lactamase (ESBL) (*bla*_TEM_, *bla*_SHV_, *bla*_CTX−M_, and *bla*_OXA−1_), plasmid-mediated AmpC *bla*_CMY−2_, and carbapenemases (*bla*_KPC_, *bla*_NDM_, *bla*_VIM_, and *bla*_OXA−48_) genes by Polymerase chain reaction. Moreover, the presence of virulence-encoding genes was investigated. The virulent MDR strains were clustered using R with the pheatmap package. The prevalence of *E. coli* was 72.7% (24 out of 33), with 66.7% of the isolates classified as multi-drug resistant, and 75% exhibited MAR values exceeding the 0.2 threshold. Different antimicrobial sensitivity phenotypes and genotype profiles were identified in *E. coli* isolates. The most prevalent gene detected among all isolates was *bla*_TEM_ (22/24, 91.7%). Notably, all non-ESBL producers were positive for *bla*_CMY2_. Carbapenem-resistant and carbapenem-intermediate strains were carbapenemase producers, with the predominance of the *bla*_KPC_ gene (11/24, 45.8%). Remarkably, twelve out of sixteen virulence genes were identified, with *papC* (21/24, 87.5%) and *sfa* (16/24, 66.7%) genes being the most prevalent. Most isolates carry virulence genes primarily associated with extra-intestinal pathogenic *E. coli* (ExPEC) (87.5%) and enteropathogenic (EPEC) (70.8%) pathotypes. Four *E. coli* isolates exhibit cluster patterns. This study provides the first insight into the emergence of virulent MDR *E. coli* among oysters in Egypt. It underscores the potential role of oysters as a source for disseminating these strains within aquatic ecosystems, presenting a possible threat to public health.

**Supplementary Information:**

The online version contains supplementary material available at 10.1038/s41598-024-77519-y.

## Introduction

The rise of intensive aquaculture has significantly contributed to the emergence and spread of antimicrobial-resistant (AMR) bacteria in marine environments, including human pathogens such as *Escherichia coli*^1^. In aquaculture systems, especially those involving bivalve mollusks like oysters, contamination with *E. coli* poses serious health risks^2^. Oysters can accumulate pathogenic bacteria and viruses from polluted waters, primarily through exposure to sewage discharge and agricultural runoff, which are significant sources of *E. coli* contamination^3,4^.

The aquatic environment is an ideal breeding ground for developing resistance among bacterial populations, which can be transmitted to humans through the food chain. This highlights the critical need for better aquaculture management to reduce the risks of antimicrobial-resistant bacteria^5^. The growing global health crisis of AMR is projected to cause 10 million deaths annually by 2050, with Asia and Africa being particularly affected^6–8^. *E. coli* plays a significant role in this crisis, harboring multiple resistance genes that complicate treatment. The misuse of antibiotics in humans and animals, along with horizontal gene transfer between bacteria, accelerates the spread of resistance^9,10^. Increasing resistance to antibiotic classes like β-lactams, fluoroquinolones, tetracyclines, and aminoglycosides has been reported globally, including in Egypt^11–14^. Understanding the genetic mechanisms driving resistance is crucial in controlling its spread^15^.

*Enterobacteriaceae* can resist beta-lactam antibiotics by producing different types of β-lactamases^16^. The most common β-lactamases are class A β-lactamases (ESBLs) and class C β-lactamases (AmpC)^17^. In contrast to ESBL, the AmpC group is not susceptible to β-lactamase inhibitors, such as clavulanic acid, sulbactam, and tazobactam^18^. The most prevalent and clinically relevant extended-spectrum beta-lactamases (ESBLs) variants are *bla*_TEM_, *bla*_SHV_, *bla*_CTX−M_, and *bla*_OXA−1_^19,20^. Meanwhile, most identified AmpC beta-lactamases have been of the CMY type^21^.

Carbapenems are considered the last-resort antibiotics for treating infections caused by multidrug-resistant gram-negative bacteria, including ESBL and AmpC-producing bacteria^22^. The emergence of carbapenem-resistant *Enterobacteriaceae* (CRE) now significantly threatens human health^1,23^. Carbapenem resistance could be inferred by a wide array of carbapenemases (CPases). In contrast, the most remarkable enzymes are *K. pneumoniae* carbapenemases (KPC), and Metallo-β-lactamases (MBL), including (VIM, NDM, IMP, SPM, GM, and SIM) types, and oxacillin-hydrolyzing metallo-β-lactamases (OXA-48)^24,25^.

According to their pathogenic characteristics, *Escherichia coli* is divided into intestinal pathogenic *E. coli* (IPEC) and extra-intestinal pathogenic *E. coli* (ExPEC). IPEC involves enteropathogenic (EPEC), enterotoxigenic (ETEC), enteroaggregative (EAEC), enteroinvasive (EIEC), and enterohemorrhagic pathotypes (EHEC)^26^, whereas ExPEC incorporates neonatal meningitis *E. coli* (NMEC), sepsis-associated *E. coli* (SEPEC), uropathogenic *E. coli* (UPEC), and avian pathogenic *E. coli* (APEC)^27^.

These pathogenic strains have originated from commensal ones by acquiring virulence-encoding genes^28^. These genes represent the main categories of virulence determinants, including adhesins (*sfa*,* papC*,* sepA*,* etrA*,* aer*,* feaG*,* fsaA*, and *eaeA*), capsule synthesis (*rfc*), and toxins (*cnf1*,* hlyA*,* eltA*,* estA*,* exhA*,* stx1*, and *stx2*)^29^.

The co-occurrence of AMR and virulence traits in *E. coli* is particularly concerning, as it increases the risk of severe, untreatable infections transmitted *via* the food chain^30–32^.

Bivalve mollusks, including oysters, play a crucial role in the aquatic ecosystem and have significant economic value as a source of human food and aquaculture industry expansion^33,34^. However, they are associated with foodborne illnesses primarily due to their filter-feeding behavior, which allows them to harbor pathogens from sewage or marine environments^35–37^. Therefore, raw consumption of oysters and other bivalves poses a potential health hazard to consumers^38^.

Although numerous studies have investigated the prevalence of pathogenic *E. coli* in food-producing animals, animal products, and human sources^39–41^, there is a gap in understanding the occurrence of pathogenic *E. coli* in marine bivalves, particularly in Egypt^2,42^. Accordingly, this study aims to address this gap by determining the prevalence of *E. coli* in oyster samples from Egypt, identifying the bacteria’s resistance and virulence gene profiles, and assessing the potential zoonotic risks associated with oyster consumption.

## Methods

### Sample collection and processing

A total of 330 fresh oysters were randomly purchased from different retail fish markets in Cairo and Giza governorates over one year, from December 2021 to December 2022. The samples were immediately transported to the laboratory under sterile refrigerated conditions and grouped into 33 pools containing ten oyster samples. Each pool corresponds to a distinct market, emphasizing one per market^1^.

The outer shells of oysters were thoroughly rinsed with sterile water to eliminate any external contaminants. Subsequently, the pooled oysters were aseptically opened, and digestive tissues were dissected, cleaned, and finely minced to form a uniform paste-like mixture^43^.

### Isolation and identification of *Escherichia coli*

Two-gram aliquots from each pool were enriched in 5 ml brain heart infusion broth (Oxoid, Hampshire, UK) for 24 h at 37 °C. After enrichment, a loopful of the broth was streaked onto MacConkey agar (Oxoid, Hampshire, UK) and Eosin methylene blue agar (EMB) (Oxoid, Hampshire, UK) and then incubated aerobically at 37 °C for 24 to 48 h. Characteristic pink color colonies on MacConkey and metallic green shiny colonies on EMB were picked from the plates and subcultured to obtain a pure culture. *E. coli* isolates were identified by colonial morphology and gram staining. Conventional biochemical tests, including the indole, methyl red, Voges-Proskauer, and citrate tests (IMViC), as well as the Triple Sugar Iron test, were used for further characterization of the bacteria, according to the methods described by Nolan^44^ and Tille^45^.

### Antimicrobial susceptibility testing (AST)

The antimicrobial sensitivity phenotypes of all confirmed *E. coli* isolates were carried out by Kirby-Bauer disc diffusion assay using commercial discs (Oxoid, Hampshire, UK) on Muller-Hinton agar (HiMedia), and the results were interpreted according to the clinical breakpoints recommended by the Clinical and Laboratory Standards Institute (CLSI)^46^. Fifteen antibiotic discs were selected based on their frequent usage in human and veterinary medicine^47^. These discs represent seven different antimicrobial classes: β-lactams (Penicillins: Ampicillin 10 µg, Cephalosporins: Cefotaxime 30 µg, Ceftazidime 30 µg, Ceftriaxone 30 µg, and Cefoxitin 30 µg, and Carbapenems: Ertapenem 10 µg and Meropenem 10 µg), Aminoglycosides (Amikacin 30 µg), Fluorquinolones (Ciprofloxacin 5 µg and Levofloxacin 5 µg), Macrolides (Erythromycin 30 µg and Azithromycin 15 µg), Tetracycline (Doxycycline 30 µg), Sulfonamides (Trimethoprim/Sulfamethoxazole 1.25 µg/23.75 µg), and Phenicols (Chloramphenicol 30 µg).

Multi-drug resistance (MDR) bacteria were defined as bacteria that are non-susceptible to at least one antimicrobial agent in three or more antimicrobial classes^48^.

The Multiple Antibiotic Resistance Index (MAR) is a valid method to track the source of bacteria^49^. MAR was calculated by dividing the number of antibiotics to which the organism is resistant by the total number of antibiotics tested^50,51^. MAR index values greater than 0.2 indicate that the isolate originated from a source where antibiotics were used to a great degree and/or in large amounts. MAR of 1.0 means that the isolate is resistant to all antibiotics tested against^52^.

An ESBL confirmatory test was performed using the Double Disc Synergy Test (DDST) employing Ceftazidime (CAZ) (30 µg), CAZ-clavulanate (30 µg/10 µg), Cefotaxime (CTX) (30 µg), and CTX-clavulanate (30 µg/10 µg) discs according to CLSI^46^.

### Extraction of the genomic DNA

Bacterial DNA was extracted from the phenotypic-resistant, intermediate, and sensitive *E. coli* isolates using a conventional boiling method, according to Ibrahim et al.^53^. The NanoDrop spectrophotometer was used to check each sample’s DNA concentration and purity. The extracted DNA was preserved at -20 °C until further use in PCR.

### Molecular detection of β-lactamase (ESBLs), AmpC-(*bla*_CMY−2_) and carbapenemase-encoding genes

#### PCR for the detection of β-lactamase-encoding genes (ESBLs)

Multiplex polymerase chain reaction (PCR) was performed to detect the resistance determinant genes *bla*_TEM_, *bla*_SHV_, *bla*_CTX−M_, and *bla*_OXA−1_ using specific oligonucleotide primer sets (Table S1). PCR was carried out on a total volume of 25 µl, containing 3 µl of template DNA from each isolate, 12.5 µl of Emerald Amp MAX PCR master mix (Takara, Japan), 0.5 µl of each primer (10 pmol/µl; Metabion, Germany), and completed up to 25 µl by PCR-grade water. The amplification conditions were as follows: 95ºC for 5 min; 30 cycles of 94ºC for 30 s, 62ºC for 90 s, and 72ºC for 60 s, followed by a final extension at 72ºC for 10 min^54,55^.

#### PCR for the detection of plasmid-mediated AmpC β-lactamase gene (*bla*_CMY−2_)

All *E. coli* isolates were subjected to uniplex PCR to determine the presence of the plasmid-mediated AmpC β-lactamase gene (*bla*_CMY−2_) as described previously by Kim et al.^56^ using specific oligonucleotide primers (Table S1). The PCR reaction mixtures of 25 µl total volume contain 12.5 µl of 2 × Emerald Amp MAX PCR master mix (Takara, Japan), 8.5 µl water, 3 µl of template DNA from each isolate, and 0.5 µl of each primer with a concentration of 20 pmol (Metabion, Germany). The PCR amplification was carried out with the following thermal profile: 94ºC for 5 min followed by 30 cycles of denaturation (94ºC for 1 min), annealing (61ºC for 1 min), extension (72ºC for 1 min), and a final extension at 72ºC for 5 min.

#### PCR for the detection of carbapenemase-encoding genes (*bla*_KPC_, *bla*_NDM_, *bla*_VIM_, and *bla*_OXA−48_)

Multiplex PCR to detect *bla*_KPC_ and *bla*_NDM_ genes was performed on all *E. coli* isolates using a specific oligonucleotide primer set (Table S1). The PCR mixtures were carried out on a total volume of 25 µl, containing 3 µl of template DNA from each isolate, 12.5 µl of Emerald Amp MAX PCR master mix (Takara, Japan), 0.5 µl of each primer (10 pmol/µl; Metabion, Germany), and completed up to 25 µl by PCR-grade water. All reaction mixtures were subjected to 30 cycles of 94ºC for 1 min, 55ºC for 1 min, 72ºC for 2 min, and a final extension at 72ºC for 10 min^57^.

An additional uniplex PCR test targeting the *bla*_VIM_ and *bla*_OXA−48_ genes was performed using specific oligonucleotide primers (Table S1). The amplification of the *bla*_VIM_ gene was performed according to Li et al.^58^. All reaction mixtures were subjected to 35 cycles of 94ºC for 30 s, 55ºC for 30 s, 72ºC for 1 min, and a final elongation at 72ºC for 10 min. Whereas the thermal cycling process of the *bla*_OXA−48_ gene consisted of 30 cycles of 94 °C for 10 min, 94 °C for 40 s, 60 °C for 40 s, 72 °C for 1 min, and final extension at 72 °C for 7 min^59^.

### Molecular determination of *Escherichia coli* virulence genes and *E. coli* pathotypes

Uniplex PCR was performed on *E. coli* isolates to detect the virulence determinants, including adhesins (*sfa*,* papC*,* sepA*,* etrA*,* aer*,* feaG*,* fsaA*, and *eaeA*), toxins (*cnf1*,* hlyA*,* eltA*,* estA*,* exhA*,* stx1*, and *stx2*), and capsule synthesis (*rfc*). The reaction was performed according to Zhao et al.^29^ and Mario et al.^41^. The PCR program consisted of an initial denaturation at 94 °C for 5 min, followed by 30 cycles of denaturation at 95 °C for 30 s, annealing at the specific temperature for each primer pair for 30 s, and extension at 72 °C for 30 s. A final extension step was performed at 72 °C for 10 min. The sequences, annealing temperatures of primers used, and amplicon sizes are outlined in Table S2.

*E. coli* strains were categorized into different pathotypes based on specific gene presence. ExPEC pathotypes express the genes *papC*, *sfa*,* cnf1*,* hlyA*,* rfc*, and *sepA*. The EAEC and EIEC pathotypes express the *etrA* and *aer* genes, respectively. ETEC expresses the genes *eltA*,* estA*,* faeG*, and *fasA.* EPEC expresses the genes *eaeA* and *exhA*, while EHEC expresses the genes *stx1* and *stx2*. Strains that tested positive for genes expressed by multiple pathotypes were classified as hybrid strains^29,41^.

All PCR products were run on 1.5% agarose gel and visualized using a UV transilluminator. A 100 bp DNA ladder (size range 100–1000 bp; Jenna Bioscience GmbH, Jenna, Germany) was run simultaneously to determine the size of the PCR amplicon. A negative control was included, containing all components of the PCR mixture but with water instead of template DNA. The positive controls were the *E. coli* strains ATCC 25,922 and ATCC 43,888.

### Statistical analysis

Statistical analysis was conducted in R (version 4.2.2, R Foundation for Statistical Computing). The strains were clustered using the pheatmap library (version 1.0.12)^60^.

## Results

**Occurrence of** ***Escherichia coli*** **in the examined sample pools**

A total of 24 confirmed *E*. *coli* isolates were identified from 33 fresh oyster pooled samples collected from different retail fish markets in Egypt, representing 72.7% of the samples.

### Antimicrobial susceptibility profile of the identified *E. coli* isolates

**Fig. 1:**
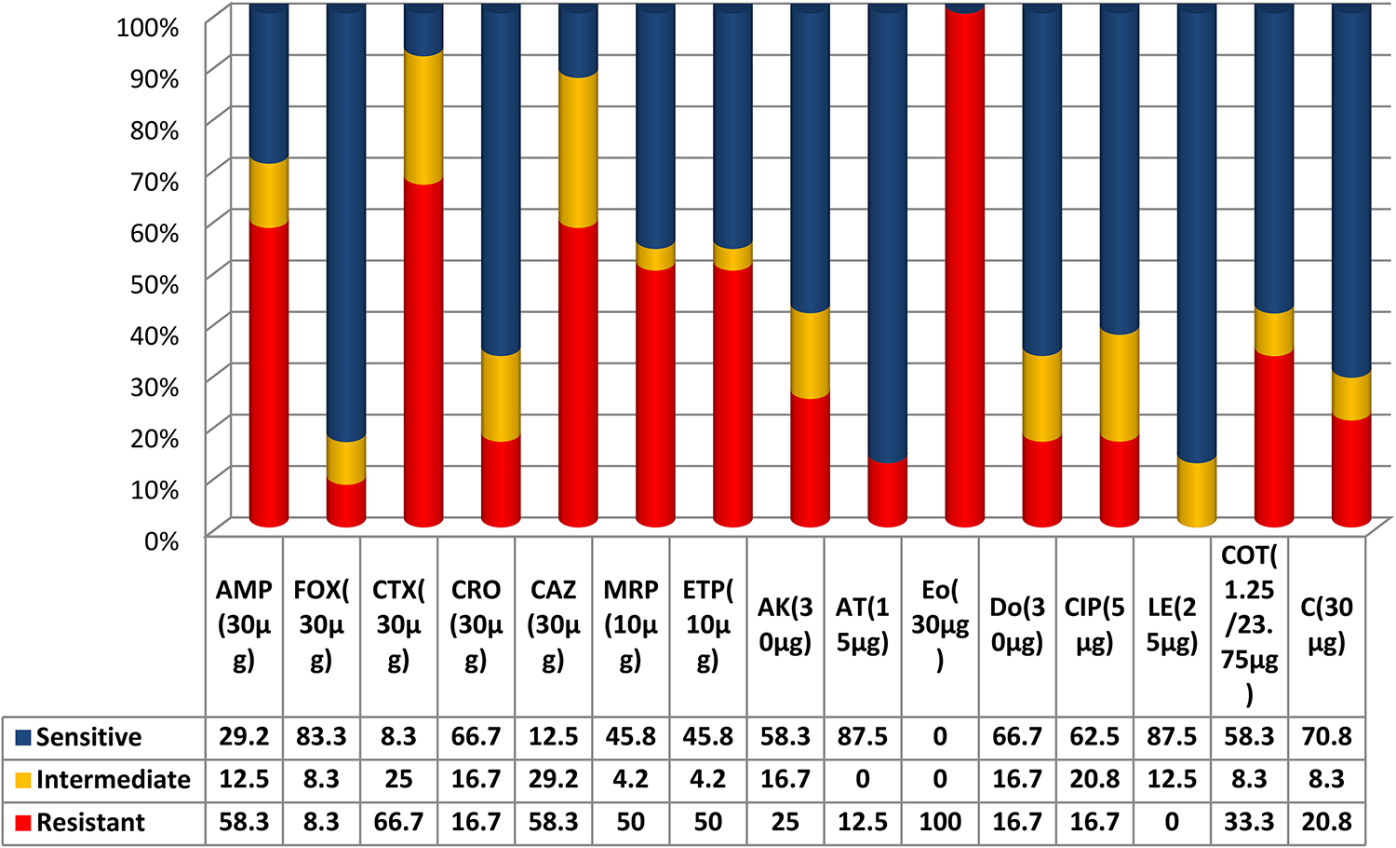
Percentage of antimicrobial resistance and intermediate resistance determined by disc diffusion method in *E. coli* isolates from retail oysters in Egypt. Antibiotic discs: Ampicillin (AMP), Cefoxitin (FOX), Cefotaxime (CTX), Ceftriaxone (CRO), Ceftazidime (CAZ), Meropenem (MRP), Ertapenem (ETP), Amikacin (AK), Azithromycin (AT), Erythromycin (Eo), Doxycycline (Do), Ciprofloxacin (CIP), Levofloxacin (LE), Trimethoprim-Sulfamethoxazole (COT), and Chloramphenicol (C).

**Fig. 2:**
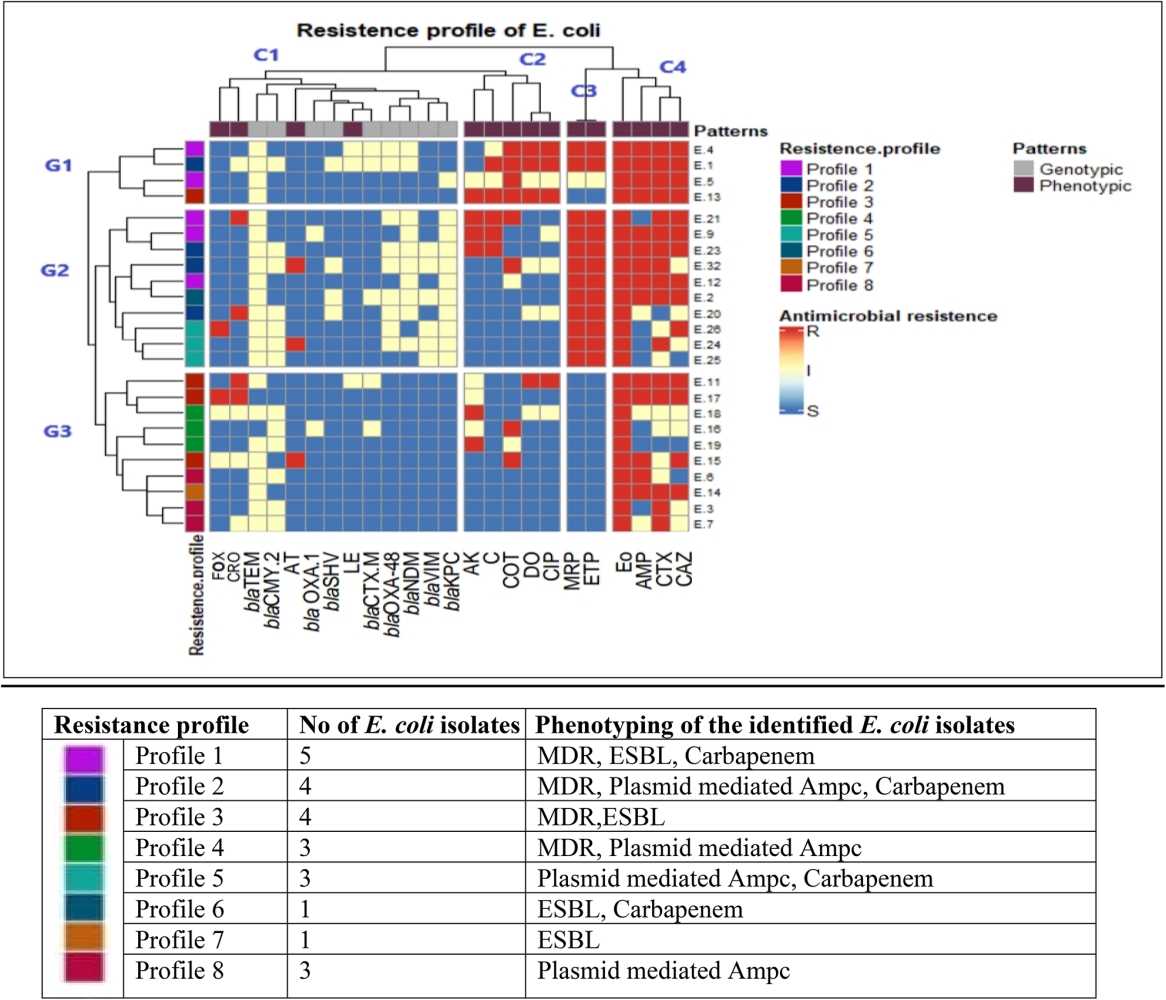
Heatmap of *E. coli* strains clustered according to both phenotypes and genotypes. G1, G2 and G3 represent the clustering of the isolates according to their antimicrobial resistance phenotype and genotype profiles, while the top of the heatmap (C1, C2, C3 and C4) represents the pattern of antibiotic resistance and resistance genes tested. The varied colors represent positive resistance genes (light yellow) or negative (blue) results, regarding the antimicrobial resistance; resistant phenotype (red), intermediate resistant (light yellow) and sensitive (blue). The phenotypic resistance patterns were screened against fifteen antibiotic discs: Ampicillin (AMP), Cefoxitin (FOX), Cefotaxime (CTX), Ceftriaxone (CRO), Ceftazidime (CAZ), Meropenem (MRP), Ertapenem (ETP), Amikacin (AK), Azithromycin (AT), Erythromycin (Eo), Doxycycline (Do), Ciprofloxacin (CIP), Levofloxacin (LE), Trimethoprim-Sulfamethoxazole (COT), and Chloramphenicol(C).The resistance genotypes are also provided; β-lactamases (*bla*_TEM_, TEM beta-lactamase; *bla*_CTX−M_, CTX-M beta-lactamase; *bla*_SHV_, SHV beta-lactamase; *bla*_OXA−1_, oxacillin-hydrolyzing β-lactamases ; *bla*_CMY−2_, plasmid-mediated AmpC β-lactamase gene), and carbapenemases (*bla*_KPC,_*K. pneumoniae* carbapenemases; *bla*_NDM,_ New Delhi metallo-beta-lactamase; *bla*_OXA−48_, oxacillin-hydrolyzing metallo-β-lactamases; *bla*_VIM_ ,Verona vntegron-encoded metallo-beta-lactamase).

The current results showed that all the isolates resisted more than one of the examined antibiotics, with 100% showing resistance against erythromycin (EO). At the same time, levofloxacin (LE) and azithromycin (AT) were the most effective antibiotics (87.5%), followed by cefoxitin (FOX) (83.3%), with other susceptibility patterns shown in Fig. 1. Furthermore, the results of the study revealed that 16 (66.7%) of the isolates were classified as multi-drug resistant (MDR), and different resistance profiles were illustrated in Fig. 2 and Table S3).

Additionally, 18 isolates (75%) displayed a multiple antibiotic resistance index (MAR) surpassing 0.2, whereas 6 isolates (25%) exhibited a MAR index below 0.2. Notably, none of the isolates exhibited an index of 1.0, as detailed in Table 1.

**Table 1:** Antibiotic resistance profiles and MDR Index of the identified *E. Coli* isolates.

*E. coli* isolates	Antimicrobial resistance phenotype^®^	No of resistant antibiotics	MAR Index
E.1	AMP, CTX, CAZ, MRP, ETP, Eo, Do, CIP, COT, C	10	0.66
E.2	AMP, CTX, CAZ, MRP, ETP, Eo	6	0.4
E.3	CTX, Eo	2	0.13
E.4	AMP, CTX, CAZ, MRP, ETP, Eo, Do, CIP, COT	9	0.6
E.5	AMP, CTX, CAZ, Eo, COT	5	0.33
E.6	AMP, Eo	2	0.13
E.7	CTX, Eo	2	0.13
E.9	AMP, CTX, CAZ, MRP, ETP, AK, Eo, C	8	0.53
E.11	AMP, CTX, CRO, CAZ, Eo, Do, CIP	7	0.46
E.12	AMP, CTX, CAZ, MRP, ETP, Eo	6	0.4
E.13	AMP, CTX, CAZ, AK, Eo, Do, CIP, COT, C	9	0.6
E.14	AMP, CTX, CAZ, Eo	4	0.26
E.15	AMP, CAZ, AT, Eo, COT	5	0.33
E.16	Eo, COT	2	0.13
E.17	AMP, FOX, CTX, CRO, CAZ, Eo	6	0.4
E.18	AK, Eo	2	0.13
E.19	AK, Eo	2	0.13
E.20	CRO, MRP, ETP, Eo	4	0.26
E.21	CTX, CRO, CAZ, MRP, ETP, AK, Eo, COT, C	9	0.6
E.23	AMP, CTX, CAZ, MRP, ETP, AK, Eo, C	8	0.53
E.24	CTX, MRP, ETP, AT, Eo	5	0.33
E.25	MRP, ETP, Eo	3	0.2
E.26	FOX, CAZ, MRP, ETP, Eo	5	0.33
E.32	AMP, CTX, MRP, ETP, AT, Eo, COT	7	0.46

*MAR index =$$\:\:\:\frac{\begin{array}{c}Number\:of\:antibiotics\:to\:whichh\:the\:isolate\:is\:resistant\:\\\:\end{array}}{\text{T}\text{h}\text{e}\:\text{t}\text{o}\text{t}\text{a}\text{l}\:\text{n}\text{u}\text{m}\text{b}\text{e}\text{r}\:\text{o}\text{f}\:\text{a}\text{n}\text{t}\text{i}\text{b}\text{i}\text{o}\text{t}\text{i}\text{c}\text{s}\:\text{t}\text{e}\text{s}\text{t}\text{e}\text{d}\:(\mathbf{n}=15)}$$

### Phenotypic and genotypic detection of ESBL and AmpC-producing *E. coli*

The Double Disc Synergy test (DDST) revealed that 11 isolates (45.8%) were ESBL-producing and 13 (54.2%) were non-ESBL-producing. Among the *E. coli* isolates, the presence of ESBL variants was tested using PCR. Out of these, 22/24 (91.7%) were positive for *bla*_TEM_, 5/24 (20.8%) for *bla*_CTX−M_, 4/24 (16.7%) for *bla*_SHV _and 2/24 (8.3%) isolates for *bla*_OXA−1_. Further, 54.2% (13 out of 24) of the isolates tested positive for *bla*_CMY−2_; notably, all were non-ESBL producers. The genotyping pattern of these β-lactamases (ESBLs) and AmpC-(*bla*_CMY−2_) genes in all *E. coli* isolates were illustrated in a heatmap (Fig. 2; Table S3).

### Phenotypic and genotypic detection of carbapenem-resistant *E. coli*

Out of 24 *E. coli* isolates tested for carbapenem susceptibility, 12 were carbapenem-resistant (50%), one was intermediate-resistant (4.2%), and 11 were sensitive (45.8%). PCR analysis targeting carbapenemase genes revealed that 45.8% carried the *bla*_KPC_ gene, 41.7% had the *bla*_NDM_ gene, 37.5% harbored *bla*_OXA−48_, and 25% contained *bla*_VIM_. All phenotypically carbapenem-resistant and intermediate *E. coli* isolates were carbapenemase producers carrying carbapenemase-encoding genes, while carbapenem-sensitive isolates lacked these genes (Fig. 2 and Table S3).

### Occurrence of *Escherichia coli* virulence genes and *E. coli* pathotypes

**Fig. 3:**
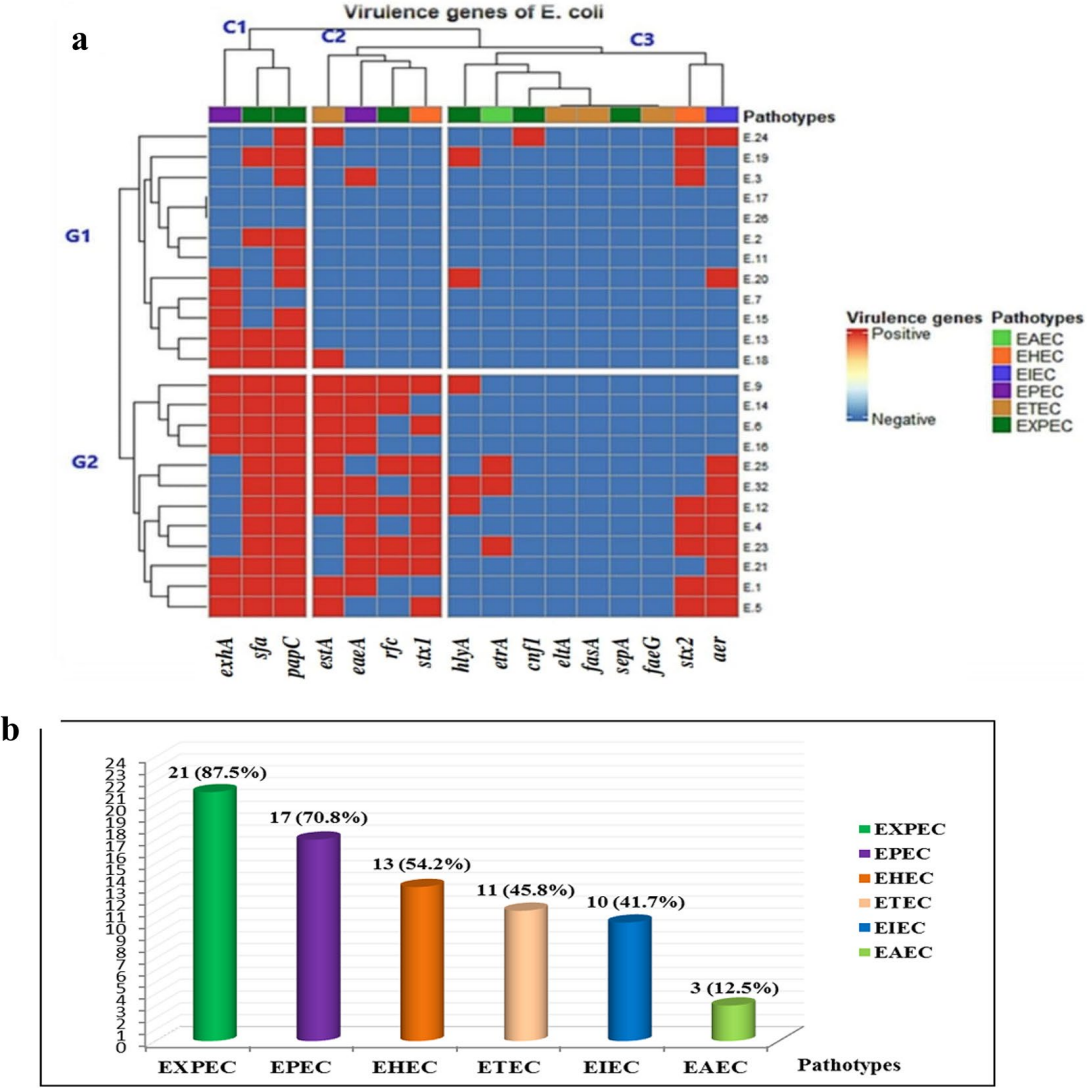
(**a**) Heatmap of *E. coli* strains clustered according to both virulence genes and their association with pathotypes. G1, G2 represent the clustering of the isolates according to their virulence genes, the top of the heatmap (C1, C2 and C3) illustrate the different pathotypes present in each isolate; (**b**) Percentage of occurrence of different pathotypes among the recovered *E. coli* isolates.

The PCR results for virulence genes in *E. coli* isolates reveal the detection of twelve out of sixteen genes. The most prevalent gene was *papC* (87.5%), followed by *sfa* (66.7%), *exhA* (50%), *eaeA* and *estA* (45.8%), *aer* (41.7%), *stx1* (37.5%), *stx2* (33.3%), *rfc* (25%), *hlyA* (20.8%), *etrA* (12.5%), and *cnf1* (4.2%). None of the isolates tested positive for *sepA*, *faeG*, *fasA*, and *eltA* genes, as displayed in Fig. 3A and Table S4.

The results show that 19 out of 24 isolates possessed a combination of virulence genes associated with different pathotypes. Notably, isolate E.32 contains virulence genes linked to six pathotypes (EXPEC, EPEC, EHEC, ETEC, EIEC, and EAEC), making it the most virulent among the isolates. Among the tested virulence genes, two isolates lacked all 16 genes, while three isolates possessed virulence gene(s) associated with only one pathotype. Overall, the majority of isolates carry virulence genes mainly related to ExPEC (87.5%) and EPEC (70.8%) pathotypes (Fig. 3A, B; Table S4).

### Cluster analysis of the antimicrobial sensitivity results with different virulence genes carriage in MDR *E. coli* isolates (*n* = 16)

**Fig. 4:**
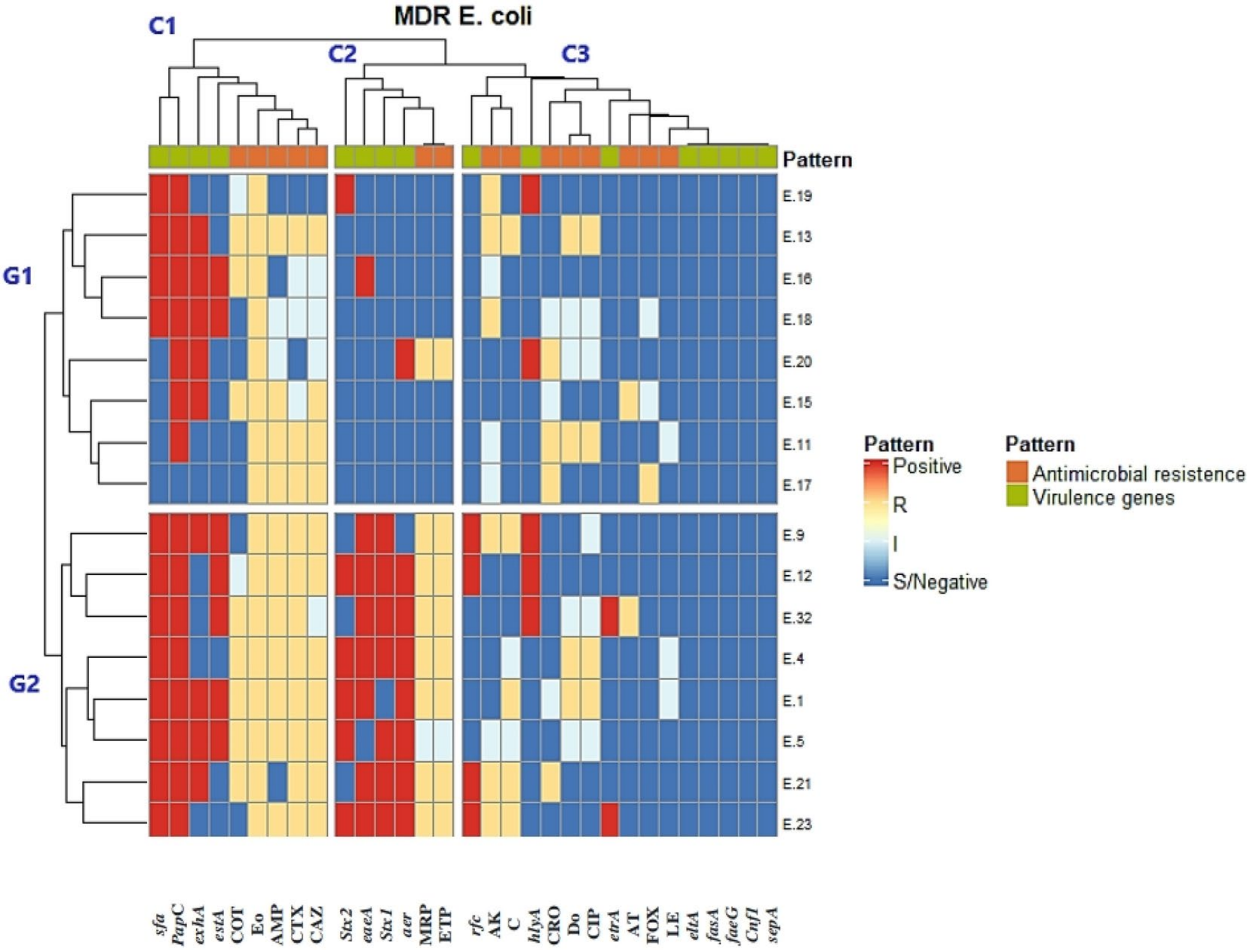
Heatmap of MDR *E. coli* isolates clustered according to both antimicrobial resistance phenotype and virulence genes carriage. The varied colors represent positive virulence genes (red) or negative (blue) results; regarding the antimicrobial resistance; resistant phenotype (yellow), intermediate resistant (light blue), and sensitive (blue). Antibiotic discs: Ampicillin (AMP), Cefoxitin (FOX), Cefotaxime (CTX), Ceftriaxone (CRO), Ceftazidime (CAZ), Meropenem (MRP), Ertapenem (ETP), Amikacin (AK), Azithromycin (AT), Erythromycin (Eo), Doxycycline (Do), Ciprofloxacin (CIP), Levofloxacin (LE), Trimethoprim-Sulfamethoxazole (COT), and Chloramphenicol(C).

The heatmap (Fig. 4) categorizes 16 MDR *E. coli* isolates into two main groups (G1 and G2) based on 15 antimicrobial resistance phenotypes and 16 virulence genes. The top of the heat map (C1, C2, and C3) shows the antibiotic resistance pattern and virulence genes tested.

In this study, 14 out of 16 (87.5%) MDR *E. coli* isolates carried more than one virulence gene. One isolate (E.11) had only the *papC* gene, while one isolate (E.17) was negative for all virulence genes. The most prevalent virulence genes among the MDR isolates were *papC* (93.8%) and *sfa* (75%). Conversely, *cnf1*,* sepA*,* faeG*,* fasA*, and *eltA* genes were absent in all isolates, as shown in Table S5.

Within the clustering, one MDR strain (E.18) exhibited a virulence profile of *estA*,* exhA*,* papC*, and *sfa*, and clustered with another isolate (E.16), sharing the presence of *estA*,* exhA*,* papC*,* sfa*, and *eaeA*. Additionally, two isolates (E.1 and E.5) had nearly identical virulence profiles, with E.1 carrying *sfa*,* papC*,* exhA*,* estA*,* stx2*,* eaeA*, and *aer*, and E.5 carrying *sfa*,* papC*,* exhA*,* estA*,* stx2*,* stx1*, and *aer*.

## Discussion

*Escherichia coli*, a prominent foodborne pathogen, presents a significant worldwide food safety and public health challenge^61^. In this study, *E. coli* was isolated from 24 out of 33 (72.7%) pooled oyster samples. This finding agrees with a study conducted on samples from the EU-imposed Norwegian surveillance program, which found that 67% of bivalves were contaminated with *E. coli*^62^. These results suggest widespread fecal contamination, potentially from sources such as human waste, animal and bird droppings, and untreated sewage discharge^4,63^. Post-harvest contamination during handling, washing, and ice storage during transportation to retail markets is highlighted^64^.

Antimicrobial resistance has been recognized as an emerging worldwide problem, and several studies have documented drug-resistant *E. coli* in aquaculture^65–67^. In the current study, 66.7% (16/24) of the isolates were classified as MDR, with 75% (18/24) having MAR values exceeding 0.2, indicating that the isolates originated from a source where antibiotics were used to a great degree and/or in large amounts^52^. Furthermore, the highest resistance was observed against erythromycin (EO) (100%). This is consistent with Dewi et al.^68^, who reported elevated resistance to EO in Asian seabass on the west coast of Peninsular Malaysia. EO has been a widely used antibiotic since the 1950s, and its common presence in the aquatic environment is due to ineffective removal by sewage treatment systems^69,70^. Additionally, it should be considered that gram-negative bacteria have intrinsic resistance to macrolides because of their outer membranes’ limited permeability, which restricts the antibiotics that may enter the cell^71^.

Moreover, phenotypical examination of isolates evidenced that 45.8% (11/24) were ESBL-producing, while 54.2% (13/24) were non-ESBL-producing. The widespread and uncontrolled use of beta-lactam antibiotics in humans and animals creates selective pressure for the emergence of ESBL-producing *E. coli*^9,72^. Notably, both phenotypically ESBL and non-ESBL-producing isolates harbored ESBL genes, primarily the *bla*_TEM_ gene. Similar findings have been documented in previous studies on retail chicken giblets and human samples from Egypt and Niger by Abdel-Kader et al.^73^ and Enyinnay et al.^74^, who found evidence of common mobile genetic elements in non-ESBL-producing *E. coli* isolates, suggesting the potential for ESBL gene transmission. However, this does not necessarily imply that these genes are functioning or being expressed^75^.

Additionally, the plasmid-mediated AmpC *bla*_CMY−2_ gene was detected in all non-ESBL isolates, while ESBL producers tested negative for this gene. This highlights the increasing global concern about plasmid-mediated AmpC enzymes in *Enterobacterales*^76–78^.

According to the Centers for Disease Control and Prevention (CDC)^79^, isolates resistant to at least one carbapenem drug or those producing carbapenemases are classified as CRE. In this study, 13 isolates were phenotypically carbapenem-resistant and carbapenem-intermediate, and all were carbapenemase producers, predominantly carrying the *bla*_KPC_ gene; this aligns with a study from Egypt that detected a high frequency of *bla*_KPC_ among *Enterobacterales* isolates from integrated fish farms^65^. These results highlight the importance of combining the resistance, phenotype, and genotype analysis when testing for AMR. Seven of these isolates were phenotypically non-ESBL-producing, while six were ESBL-producing. Similar findings have been documented in a study on human samples from Pakistan by Mustafai et al.^80^, who reported carbapenem resistance rates of 31.4% for non-ESBL-producing *E. coli* and 39.2% for ESBL-producing *E. coli.* The presence of carbapenemase genes in ESBL-producing and non-ESBL-producing isolates highlights the complexity of antibiotic resistance mechanisms^81,82^. The overproduction of AmpC and/or ESBLs can lead to the convergence of carbapenemase resistance phenotypes^83–85^. Additionally, competitive inhibition among these enzymes may reduce their efficacy^86^.

The current study highlights variations in the antibiotic susceptibility patterns of MDR strains. Notably, carbapenem resistance was detected along with ESBLs or plasmid-mediated AmpC. Furthermore, some MDR strains exhibited either ESBLs or plasmid-mediated AmpC enzymes. The production of β-lactamase enzymes can contribute to multi-drug resistance in *E. coli*^87^. These enzymes are encoded by genes found on plasmids or single bacterial chromosomes, which harbor genes responsible for resistance to other antimicrobial agents^88^. The emergence of dual-resistant strains is primarily attributed to plasmid-mediated gene transfer between related and unrelated bacterial species, as suggested by Sun et al.^10^.

*E. coli* has an array of virulence genes that are a key factor for causing intestinal and extra-intestinal disease, attracting global research interest. In this study, 12 out of 16 virulence genes were detected in *E. coli* isolates, indicating that oysters may harbor pathogenic *E. coli* strains with abundant and diverse virulence factors, which are a significant public health threat since such strains could be transmitted to humans either through direct contact or the food chain^30^.

The most common genes among *E. coli* virulence genes in this study were *papC* and *sfa*. Such observations are aligned with Mario et al.^41^, who discovered the widespread presence of the *PapC* gene in *E. coli* isolates from animals, and Cunha et al.^89^ observed elevated frequencies of *sfa* and *papC* genes in various poultry farms across Brazil. Notably, the pilus associated with pyelonephritis *(papC)* and S fimbriae sialic acid-specific (*sfa*) genes may play a significant role in the bacterial ability to colonize and infect the cells of the gut and other organs, leading to tissue damage and disease in humans^27,29^.

The current results showed that many *E. coli* isolates from oysters carried virulence genes associated with EXPEC and EPEC pathotypes. This aligns with findings by Hamelin et al.^90^, who reported that most *E. coli* isolated from aquatic ecosystems within the St. Clair River and Detroit River Areas were ExPEC pathotypes. Furthermore, the EPEC pathotype has been identified in shellfish in Brazil^91^, as well as in oysters and hard clams in Venezuela^92^, and in oysters, mussels, and cockles in France^93,94^. Notably, the majority of *E. coli* isolates obtained from humans also belong to the EPEC and ExPEC pathotypes^95,96^, which aligns with our findings in oysters. These results enhance our understanding of *E. coli* pathotypes and their potential to infect both humans and animals.

Alarmingly, most of the recovered isolates contain virulence genes linked to more than one pathotype. This can be explained by the frequent gains and losses of genes between various bacterial species through horizontal gene transfer, resulting in the diversification of new strains and sometimes “hybrid” pathotypes^97^. Thus, characterizing strains with combinations of virulence traits becomes crucial in defining their pathogenic potential^98^.

To investigate the diversity and evolution of the pathogen, MDR *E. coli* isolates were categorized based on their antimicrobial resistance phenotype and virulence genes using the pheatmap library. Notably, isolates (E.1, E.5) and (E.16, E.18) exhibited nearly identical virulence profiles. This similarity suggests a common source, possibly contaminated water, handling practices, or processing facilities. Additionally, these isolates may have typical suppliers or geographic origins, contributing to similar microbial populations, as indicated by studies by Chen et al.^99^ and Yu et al.^100^.

Addressing the presence of multiple antibiotic resistances with similar virulence features in Egyptian oysters is a significant challenge for treating infectious diseases in humans and animals. The accumulation of these strains in oysters, possibly acquired from water and processing, suggests a potential public health crisis through the food chain and emphasizes the marine environment as a source of critical bacterial pathogens.

## Conclusion

This study highlights the role of marine bivalves, particularly oysters, in spreading virulent multi-drug-resistant *E. coli*, including resistance to newer antibiotics like carbapenems. Variations in antibiotic susceptibility suggest aquatic environments as hotspots for resistance genes, with high pathogenicity confirmed by virulence assays. Limitations include sample pooling variability and a small number of isolates analyzed by PCR, potentially missing rare resistance or virulence traits; however, this allows for a more focused analysis of each isolate. Future studies with larger samples and advanced genomic techniques are needed. The findings emphasize the importance of food safety, responsible antibiotic use, and global collaboration to protect public health, particularly seafood consumers.

## Electronic supplementary material

Below is the link to the electronic supplementary material.

Supplementary Material 1

## Data Availability

All the data generated or analyzed in this study are included in this published article.

## References

[1] Freire, S. et al. ESBL-and carbapenemase-producing Escherichia coli and Klebsiella pneumoniae among bivalves from Portuguese shellfish production areas. *Microorganisms*. **11**, 415 (2023). 36838380 10.3390/microorganisms11020415 PMC9965403

[2] Al Qabili, D. M. A., Aboueisha, A. M., Ibrahim, G. A., Youssef, A. I. & El-Mahallawy, H. S. Virulence and antimicrobial-resistance of shiga toxin-producing E. Coli (STEC) isolated from edible shellfish and its public health significance. *Arch. Microbiol.* **204**, 510 (2022). 35864384 10.1007/s00203-022-03114-2 PMC9304054

[3] Vignaroli, C. et al. New sequence types and multidrug resistance among pathogenic Escherichia coli isolates from coastal marine sediments. *Appl. Environ. Microbiol.* **78**(11), 3916–3922 (2012). 22447595 10.1128/AEM.07820-11 PMC3346399

[4] Korajkic, A. et al. Extended persistence of general and cattle-associated fecal indicators in marine and freshwater environment. *Sci. Total Environ.* **650**, 1292–1302 (2019). 30308816 10.1016/j.scitotenv.2018.09.108 PMC8982556

[5] Milijasevic, M., Veskovic-Moracanin, S., Milijasevic, J. B., Petrovic, J. & Nastasijevic, I. *Antimicrob. Resist. Aquaculture: Risk Mitigation within One Health Context Foods* 13 (2024). 10.3390/foods13152448 PMC11311770 39123639

[6] Murray, C. J. et al. Global burden of bacterial antimicrobial resistance in 2019: a systematic analysis. *Lancet*. **399**, 629–655 (2022). 35065702 10.1016/S0140-6736(21)02724-0 PMC8841637

[7] Safain, K. S. et al. Situation of antibiotic resuistance in Bangladesh and its association with resistance genes for horizontal transfer. *BioRxiv*. 2020-04 (2020).

[8] Romandini, A. et al. Antibiotic resistance in pediatric infections: global emerging threats predicting the near future. *Antibiotics*. **10**, 393–412 (2021). 33917430 10.3390/antibiotics10040393 PMC8067449

[9] Nadella, R. K. et al. Antibiotic resistance of culturable heterotrophic bacteria isolated from shrimp (*Penaeus vannamei*) aquaculture ponds. *Mar. Pollut Bull.* **172**, 112887 (2021). 34450408 10.1016/j.marpolbul.2021.112887

[10] Sun, D., Sun, X., Hu, Y., &Yamaichi, Y. & Editorial Horizontal gene transfer mediated bacterial antibiotic resistance, II. *Front. Microbiol.* **14**, 1221606 (2023). 37425999 10.3389/fmicb.2023.1221606 PMC10327596

[11] Flament-Simon, S. C. et al. Clonal structure, virulence factor-encoding genes and antibiotic resistance of Escherichia coli, causing urinary tract infections and other extraintestinal infections in humans in Spain and France during 2016. *Antibiotics*. **9**, 161 (2020). 32260467 10.3390/antibiotics9040161 PMC7235800

[12] Masoud, S. M., El-Baky, A., Mahmoud, R., Aly, S. A. & Ibrahem, R. A. Co-existence of certain ESBLs, MBLs and plasmid mediated quinolone resistance genes among MDR E. Coli isolated from diferent clinical specimens in Egypt. *Antibiotics*. **10**, 835 (2021). 34356756 10.3390/antibiotics10070835 PMC8300665

[13] Cho, S., Jackson, C. R. & Frye, J. G. Freshwater environment as a reservoir of extended-spectrum β-lactamase-producing Enterobacteriaceae. *J. Appl. Microbiol.* **134**, lxad034 (2023). 36806844 10.1093/jambio/lxad034

[14] Hinthong, W. et al. Antimicrobial resistance, virulence profile, and genetic analysis of ESBL-producing Escherichia coli isolated from Nile tilapia in fresh markets and supermarkets in Thailand. *Plos One*. **19**, e0296857 (2024). 38215169 10.1371/journal.pone.0296857 PMC10786378

[15] Deter, H. S., Hossain, T. & Butzin, N. C. Antibiotic tolerance is associated with a broad and complex transcriptional response in *E. Coli*. *Sci. Rep.* **11**, 6112 (2021). 33731833 10.1038/s41598-021-85509-7 PMC7969968

[16] Fernandez, B. et al. Mechanisms of antibiotic resistance in Pseudomonas aeruginosa biofilms. *Biofilm*. **5**, 100129 (2023). 37205903 10.1016/j.bioflm.2023.100129 PMC10189392

[17] Doddaiah, V. & Anjaneya, D. Prevalence of ESBL, AmpC and carbapenemase among gram negative bacilli isolated from clinical specimens. *Amer J. Life Sci.* **2**, 76–81 (2014).

[18] Meini, S., Tascini, C., Cei, M., Sozio, E. & Rossolini, G. M. AmpC β-lactamaseproducing enterobacterales: what a clinician should know. *Infection*. **47**, 363–375 (2019). 30840201 10.1007/s15010-019-01291-9

[19] Miao, Z., Li, S., Wang, L., Song, W. & Zhou, Y. Antimicrobial resistance and molecular epidemiology of esbl-producing *Escherichia coli* isolated from outpatients in town hospitals of Shandong Province, China. *Front. Microbiol.* **8**, 63 (2017). 28174570 10.3389/fmicb.2017.00063 PMC5258711

[20] Castanheira, M., Simner, P. J. & Bradford, P. A. Extended-spectrum β-lactamases: an update on their characteristics, epidemiology and detection. *JAC Antimicrob. Resist.* **3**, dlab092 (2021). 34286272 10.1093/jacamr/dlab092 PMC8284625

[21] Madec, J. Y., Haenni, M., Nordmann, P. & Poirel, L. Extended-spectrum β-lactamase/AmpC- and carbapenemase-producing *Enterobacteriaceae* in animals: a threat for humans? *Clin. Microbiol. Infect.* **23**, 826–833 (2017). 28143782 10.1016/j.cmi.2017.01.013

[22] Taggar, G., Rehman, M. A., Boerlin, P. & Diarra, M. S. Molecular epidemiology of carbapenemases in *Enterobacteriales* from humans, animals, food and the environment. *Antibiotics*. **9**, 693 (2020). 33066205 10.3390/antibiotics9100693 PMC7602032

[23] Varandas, S. et al. Escherichia coli phylogenetic and antimicrobial pattern as an Indicator of anthropogenic impact on threatened freshwater mussels. *Antibiotics*. **12**, 1401 (2023). 37760699 10.3390/antibiotics12091401 PMC10525238

[24] Nordmann, P., Naas, T. & Poirel, L. Global spread of carbapenemase-producing Enterobacteriaceae. *Emerg. Infect. Dis.* **17**, 1791–1798 (2011). 22000347 10.3201/eid1710.110655 PMC3310682

[25] Lutgring, J. D. Carbapenem-resistant Enterobacteriaceae: an emerging bacterial threat. In *Seminars in Diagnostic Pathology*. **36**, 182–186 (2019). 10.1053/j.semdp.2019.04.011 31056277

[26] Guimarães, A. et al. Antibacterial activity of terpenes and terpenoids present in essential oils. *Molecules*. **24**, 2471 (2019). 31284397 10.3390/molecules24132471 PMC6651100

[27] Denamur, E., Clermont, O., Bonacorsi, S. & Gordon, D. The population genetics of pathogenic Escherichia coli. *Nat. Rev. Microbiol.* **19**, 37–54 (2021). 32826992 10.1038/s41579-020-0416-x

[28] Sobhy, N. M. et al. Virulence factors and antibiograms of Escherichia coli isolated from diarrheic calves of Egyptian cattle and water bufaloes. *PLoS ONE*. **15**, e0232890 (2020). 32392237 10.1371/journal.pone.0232890 PMC7213691

[29] Zhao, X. et al. Comparison of antimicrobial resistance, virulence genes, phylogroups, and bioflm formation of Escherichia coli isolated from intensive farming and free-range sheep. *Front. Microbiol.* **12**, 699927 (2021). 34394043 10.3389/fmicb.2021.699927 PMC8362090

[30] Madec, J., Haenni, M., Métayer, V., Saras, E. & Nicolas-Chanoine, M. High prevalence of the animal-associated bla CTX-M-1 IncI1/ST3 plasmid in human *Escherichia coli* isolates. *Antimicrob. Agents Chemother.* **59**, 5860–5861 (2015). 26124170 10.1128/AAC.00819-15 PMC4538533

[31] Mujeeb, U. R. et al. Experimental mouse lethality of *Escherichia coli* strains isolated from free ranging tibetan yaks. *Microb. Pathog*. **109**, 15–19 (2017). 28506886 10.1016/j.micpath.2017.05.020

[32] Araby, E., Nada, H. G., El-Nour, A., Salwa, A. & Hammad, A. Detection of tetracycline and streptomycin in beef tissues using Charm II, isolation of relevant resistant bacteria and control their resistance by gamma radiation. *BMC Microbiol.* **20**, 1–11 (2020). 32600267 10.1186/s12866-020-01868-7 PMC7325294

[33] Onada, O. A. & Ogunola, O. S. Effects of catfish (Clarias gariepinus) brood-stocks egg combination on hatchability and survival of fish larvae. *JARD*. **2**, 1–5 (2017).

[34] Cravo, A. et al. Unravelling the effects of treated wastewater discharges on the water quality in a coastal lagoon system (Ria Formosa, South Portugal): relevance of hydrodynamic conditions. *Mar. Pollut Bull.* **174**, 113296 (2022). 34995889 10.1016/j.marpolbul.2021.113296

[35] Centers for Disease Control and Prevention & Atlanta *Antibiotic Resistance Threats in the United States* (CDC, 2019). https://ndc.services.cdc.gov/wp-content/uploads/Antibiotic-Resistance-Threats-in-the-United-States-

[36] Fiorito, F. et al. Oyster *Crassostrea gigas*, a good model for correlating viral and chemical contamination in the marine environment. *Mar. Pollut Bull.* **172**, 112825 (2021). 34388447 10.1016/j.marpolbul.2021.112825

[37] Bueris, V. et al. Convergence of virulence and resistance in international clones of WHO critical priority enterobacterales isolated from Marine bivalves. *Sci. Rep.* **12**, 5707 (2022). 35383231 10.1038/s41598-022-09598-8 PMC8983722

[38] Ghribi, F. et al. Nutritional quality traits of raw and cooked Ark shell (Bivalvia: Arcidae): balancing the benefits and risks of seafood consumption. *J. Food Sci. Technol.* **58**, 3346–3356 (2021). 34366452 10.1007/s13197-020-04905-5 PMC8292546

[39] Dorado-García, A. et al. Molecular relatedness of ESBL/AmpC-producing *Escherichia coli* from humans, animals, food and the environment: a pooled analysis. *J. Antimicrob. Chemother.***.73**, 339–347 (2018). 29165596 10.1093/jac/dkx397

[40] Ramos, S. et al. *Escherichia coli* as commensal and pathogenic bacteria among food-producing animals: Health implications of extended spectrum β-lactamase (ESBL) production. *Animals*. **10**, 2239 (2020). 33260303 10.3390/ani10122239 PMC7761174

[41] Mario, E., Hamza, D. & Abdel-Moein, K. The Burden of Escherichia coli Pathotypes among Diarrheic Farm animals: a possible zoonotic relevance. *J. Adv. Vet. Res.* **13**, 1376–1380 (2023).

[42] Abdelmgeed, H. A. A. Seafood a potential source of some zoonotic bacteria in Zagazig, Egypt, with the molecular detection of Listeria monocytogenes virulence genes. *Vet. Ital.* **49**, 299–308 (2013). 24166481 10.12834/VetIt.1305.05

[43] Plante, D., Bran Barrera, J. A., Lord, M., Iugovaz, I. & Nasheri, N. Development of an RNA extraction protocol for Norovirus from raw oysters and detection by qRT-PCR and droplet-digital RT-PCR. *Foods*. **10**, 1804 (2021). 34441580 10.3390/foods10081804 PMC8393641

[44] Nolan, L., Barnes, H., Vaillancourt, J., Abdul-Aziz, T. & Logue, C. In *Diseases of Poultry*. 13th edn (eds Swayne, D. E.) (Wiley-Blackwell, 2013).

[45] Tille, P. M. *Bailey & Scott’s. Diagnostic Microbiology* 14th edn (Elsevier, 2017).

[46] CLSI, Clinical and Laboratory Standards Institute. *Performance Standards for Antimicrobial Susceptibility Testing. CLSI Supplement M100* 31th edn (CLSI, 2021).

[47] Ateba, C. N., Tabi, N. M., Fri, J., Bissong, M. E. A. & Bezuidenhout, C. C. Occurrence of antibiotic-resistant bacteria and genes in two drinking Water treatment and distribution systems in the North-West Province of South Africa. *Antibiotics*. **9**, 745 (2020). 33126462 10.3390/antibiotics9110745 PMC7692212

[48] Magiorakos, A. P. et al. Multidrug-resistant, extensively drug-resistant and pandrug-resistant bacteria: an international expert proposal for interim standard definitions for acquired resistance. *CMI*. **18**, 268–281 (2012). 21793988 10.1111/j.1469-0691.2011.03570.x

[49] Adzitey, F. Antibiotic resistance of Escherichia coli isolated from beef and its related samples in Techiman Municipality of Ghana. *Asian J. Anim. Sci.* **9**, 233–240 (2015).

[50] Ghoneim, N. H., Sabry, M. A., Ahmed, Z. S. & Elshafiee, E. A. Campylobacter species isolated from chickens in Egypt: Molecular Epidemiology and Antimicrobial Resistance. *Pak J. Zool.* **52**, 917–926 (2020).

[51] Igbinosa, I. H. et al. Identification and characterization of MDR virulent Salmonella spp isolated from smallholder poultry production environment in Edo and Delta States, Nigeria. *PLoS ONE*. **18**, e0281329 (2023). 36735693 10.1371/journal.pone.0281329 PMC9897568

[52] Mthembu, T. P., Zishiri, O. T. & El Zowalaty, M. E. Molecular detection of multidrug-resistant Salmonella isolated from livestock production systems in South Africa. *Infect. Drug Resist.* **12**, 3537–3548 (2019). 31814742 10.2147/IDR.S211618 PMC6861519

[53] Ibrahim, D. R., Dodd, C. E. R., Stekel, D. J., Ramsden, S. J. & Hobman, J. L. Multidrug resistant, extended spectrum β-lactamase (ESBL)-producing Escherichia coli isolated from a dairy farm. *FEMS Microbiol. Ecol.* **92**, fiw013 (2016). 26850161 10.1093/femsec/fiw013

[54] Monstein, H. J. et al. Multiplex PCR amplification assay for the detection of blaSHV, blaTEM and blaCTX-M genes in Enterobacteriaceae. *Apmis*. **115**, 1400–1408 (2007). 18184411 10.1111/j.1600-0463.2007.00722.x

[55] Djeffal, S. et al. Prevalence and clonal relationship of ESBL-producing Salmonella strains from humans and poultry in northeastern Algeria. *BMC Vet. Res.* **13**, 1–9 (2017). 28506272 10.1186/s12917-017-1050-3 PMC5433073

[56] Kim, J. et al. Rapid detection of extended spectrum β-lactamase (ESBL) for Enterobacteriaceae by use of a multiplex PCR-based method. *Infect. Chemother.* **41**, 181–184 (2009).

[57] Li, B. et al. Analysis of drug resistance determinants in Klebsiella pneumoniae isolates from a tertiary-care hospital in Beijing, China. *PLoS One* **7**, e42280 (2012). 10.1371/journal.pone.0042280 PMC3409176 22860106

[58] Li, J., Hu, Z. & Hu, Q. Isolation of the first IMP-4 metallo-β-lactamase producing Klebsiella pneumoniae in Tianjin, China. *J. Microbiol.* **43**, 917–922 (2012). 24031907 10.1590/S1517-838220120003000010 PMC3768900

[59] Dallenne, C., Da Costa, A., Decré, D., Favier, C. & Arlet, G. Development of a set of multiplex PCR assays for the detection of genes encoding important β-lactamases in Enterobacteriaceae. *J. Antimicrob. Chemother.* **65**, 490–495 (2010). 20071363 10.1093/jac/dkp498

[60] Kolde, R. & Pheatmap Pretty Heatmaps. R package version 1.0. 12. https://CRAN.R-project.org/package=pheatmap (2019).

[61] Abebe, E., Gugsa, G. & Ahmed, M. Review on major food-borne zoonotic bacterial pathogens. *J. Trop. Med.* **2020** (1), 4674235 (2020). 32684938 10.1155/2020/4674235 PMC7341400

[62] Svanevik, C. S., Norström, M., Lunestad, B. T., Slettemeås, J. S. & Urdahl, A. M. From tide to table: a whole-year, coastal-wide surveillance of antimicrobial resistance in Escherichia coli from Marine bivalves. *Int. J. Food Microbiol.* **407**, 110422 (2023). 37804775 10.1016/j.ijfoodmicro.2023.110422

[63] Meerburg, B. G., Koene, M. G. & Kleijn, D. Escherichia coli concentrations in feces of geese, coots, and gulls residing on recreational water in the Netherlands. *Vector-Borne Zoonotic Dis.* **11** (6), 601–603 (2011). 21548761 10.1089/vbz.2010.0218

[64] Ogur, S. Pathogenic bacteria load and safety of retail marine fish. *Braz J. Biol.* **82** (2022). 10.1590/1519-6984.262735 35792730

[65] Hamza, D. et al. Emergence of β-lactamase-and carbapenemase-producing Enterobacteriaceae at integrated fish farms. *Antimicrob. Resist. Infect. Control*. **9**, 1–12 (2020). 32430083 10.1186/s13756-020-00736-3 PMC7236517

[66] Singh, A. S., Nayak, B. B. & Kumar, S. H. HHigh prevalence of multiple antibiotic-resistant, extended-spectrum β-Lactamase (ESBL)-producing Escherichia coli in fresh seafood sold in retail markets of Mumbai, India. *Vet. Sci.* **7**, 46 (2020). 32316123 10.3390/vetsci7020046 PMC7356741

[67] Sola, M. et al. Prevalence and characterization of extended-spectrum β-Lactamase-and carbapenemase-producing enterobacterales from Tunisian seafood. *Microorganisms*. **10**, 1364 (2022). 35889085 10.3390/microorganisms10071364 PMC9323973

[68] Dewi, R. R. et al. Prevalence and antimicrobial resistance of Escherichia coli, Salmonella and Vibrio derived from farm-raised Red Hybrid Tilapia (Oreochromis spp.) and Asian Sea Bass (Lates calcarifer, Bloch 1970) on the west coast of Peninsular Malaysia. *Antibiotics*. **11**, 136 (2022). Hassan L, Daud H M, Matori MF, Nordin F, Ahmad NI, Zakaria Z, et al. 35203739 10.3390/antibiotics11020136 PMC8868497

[69] Gao, L. et al. Occurrence of antibiotics in eight sewage treatment plants in Beijing, China. *Chemosphere*. **86**, 665–671 (2012). 22154158 10.1016/j.chemosphere.2011.11.019

[70] Johnson, A. C., Keller, V., Dumont, E. & Sumpter, J. P. Assessing the concentrations and risks of toxicity from the antibiotics ciprofloxacin, sulfamethoxazole, trimethoprim and erythromycin in European rivers. *Sci. Total Environ.* **511**, 747–755 (2015). 25617699 10.1016/j.scitotenv.2014.12.055

[71] Ma, Y., Pirolo, M., Jana, B., Mebus, V. H. & Guardabassi, L. The intrinsic macrolide resistome of Escherichia coli. *Antimicrob. Agents Chemother.* **68**, e00452–e00424 (2024). 38940570 10.1128/aac.00452-24 PMC11304742

[72] Thakuria, B. & Lahon, K. The beta lactam antibiotics as an empirical therapy in a developing country: an update on their current status and recommendations to counter the resistance against them. *JCDR*. **7**, 1207–1214 (2013). 23905143 10.7860/JCDR/2013/5239.3052 PMC3708238

[73] Abdel-Kader, F., Hamza, E., Abdel-Moein, K. A. & Sabry, M. A. Retail chicken giblets contaminated with extended-spectrum cephalosporin- and carbapenem-resistant *Salmonella enterica* carrying *bla*CMY-2. *Vet. World*. **15**, 1297–1304 (2022). 35765473 10.14202/vetworld.2022.1297-1304 PMC9210848

[74] Enyinnaya, S. O., Iregbu, K. C., Jamal, W. Y. & Rotimi, V. O. Antibiotic susceptibility profiles and detection of genes mediating extended-spectrum β-lactamase (Esbl) production in escherichia coli isolates from National Hospital, Abuja. Niger. *J. Clin. Pract.* **25**, 1216–1220 (2022). 10.4103/njcp.njcp_1390_21 35975366

[75] Wilson, H. & Török, M. E. Extended-spectrum β-lactamase-producing and carbapenemase-producing Enterobacteriaceae. *Microb. Genom*. **4**, e000197 (2018). 30035710 10.1099/mgen.0.000197 PMC6113871

[76] Sen, K. et al. Antibiotic resistance of E. Coli isolated from a constructed wetland dominated by a crow roost, with emphasis on ESBL and AmpC containing E. Coli. *Front. Microbiol.* **10**, 1034 (2019). 31156579 10.3389/fmicb.2019.01034 PMC6530415

[77] Vounba, P., Arsenault, J., Bada-Alambedji, R. & Fairbrother, J. M. Antimicrobial resistance and potential pathogenicity of Escherichia coli isolates from healthy broilers in Québec, Canada. *Microb. Drug Resist.* **25**, 1111–1121 (2019). 31038391 10.1089/mdr.2018.0403

[78] Nakayama, T., Kumeda, Y., Kawahara, R. & Yamamoto, Y. Quantification and long-term carriage study of human extended-spectrum/AmpC β-lactamase-producing Escherichia coli after international travel to Vietnam. *JGAR*. **21**, 229–234 (2020). 10.1016/j.jgar.2019.11.001 31726236

[79] Centers for Disease Control and Prevention & Atlanta, A. A. M. R. C. D. C. GA, USA. https://www.cdc.gov/nhsn/pdfs/datastat-au-report-508.pdf (2020).

[80] Mustafai, M. M. et al. Prevalence of carbapenemase and extended-spectrum β-Lactamase producing *Enterobacteriaceae*: a cross-sectional study. *Antibiot. (Basel)*. **12**, 148 (2023). 10.3390/antibiotics12010148 PMC9854900 36671350

[81] Irfan, S., Azhar, A., Bashir, A., Ahmed, S. & Haque, A. High frequency of simultaneous presence of ESBL and carbapenemase producers among nosocomial coliform isolates in Faisalabad. *Pakistan Pak J. Med. Sci.* **37**, 34–39 (2021). 33437247 10.12669/pjms.37.1.3192 PMC7794153

[82] Tseng, C. H. et al. Insight into the Mechanisms of carbapenem resistance in Klebsiella pneumoniae: a study on IS26 integrons, beta-lactamases, porin modifications, and plasmidome analysis.*Antibiotics*. **12**, 749 (2023). 10.3390/antibiotics12040749 PMC10135210 37107111

[83] Ayoub Moubareck, C. & Hammoudi Halat, D. Insights into Acinetobacter baumannii: a review of microbiological, virulence, and resistance traits in a threatening nosocomial pathogen. *Antibiotics*. **9**, 119 (2020). 32178356 10.3390/antibiotics9030119 PMC7148516

[84] Karlsson, M. et al. Molecular characterization of carbapenem-resistant Enterobacterales collected in the United States. *Microb. Drug Resist.* **28**, 389–397 (2022). 35172110 10.1089/mdr.2021.0106

[85] Black, C. A. et al. Diverse role of blaCTX-M and porins in mediating ertapenem resistance among carbapenem-resistant Enterobacterales. *Antibiotics*. **13**, 185 (2024). 38391571 10.3390/antibiotics13020185 PMC10885879

[86] Veeraraghavan, B. et al. Newer β-Lactam/β-Lactamase inhibitor for multidrug-resistant gram-negative infections: challenges, implications and surveillance strategy for India. *Ind. J. Med. Microbiol.* **36**, 334–343 (2018). 10.4103/ijmm.IJMM_18_326 30429384

[87] Xexaki, A. et al. Prevalence of antibiotic resistant *E. Coli* strains isolated from farmed broilers and hens in Greece, based on phenotypic and molecular analyses. *Sustainability*. **15**, 9421 (2023).

[88] Haddadin, R. N., Collier, P. J. & Haddadin, S. Phenotypic ESBL and non-phenotypic ESBL isolates of *Klebsiella pneumoniae* exhibit differing responses to induced antimicrobials resistance and subsequent antibiotic cross-resistance. *J. Appl. Microbiol.* **134**, lxac082 (2023). 36724268 10.1093/jambio/lxac082

[89] Cunha, M. P. V. et al. Pandemic extra-intestinal pathogenic Escherichia coli (ExPEC) clonal group O6-B2-ST73 as a cause of avian colibacillosis in Brazil. *PLoS One*. **12**, e0178970 (2017). 28594893 10.1371/journal.pone.0178970 PMC5464619

[90] Hamelin, K. et al. Occurrence of virulence and antimicrobial resistance genes in Escherichia coli isolates from different aquatic ecosystems within the St. Clair River and Detroit River areas. *Appl. Environ. Microbiol.* **73**, 477–484 (2007). 17085696 10.1128/AEM.01445-06 PMC1796988

[91] Vásquez-García, A. et al. Escherichia coli detection and identification in shellfish from southeastern Brazil. *Aquaculture*. **504**, 158–163 (2019).

[92] Martínez, R. E. N. & Villalobos, L. B. B. Escherichia coli enteropatogena en moluscos crudos y cocidos. *Rev. Científica*. **15**, 163–167 (2005).

[93] Balière, C. et al. Prevalence and characterization of Shiga toxin producing and enteropathogenic Escherichia coli in shellfish-harvesting areas and their watersheds. *Front. Microbiol.* **6**, 1–15 (2015). 26648928 10.3389/fmicb.2015.01356 PMC4664706

[94] Balière, C., Rincé, A., Delannoy, S., Fach, P. & Gourmelon, M. Molecular profiling of STEC and EPEC strains isolated from French coastal environments. *Appl. Environ. Microbiol.* **82**, 3913–3927 (2016). 27107119 10.1128/AEM.00271-16 PMC4907183

[95] Manges, A. R. et al. Global extraintestinal pathogenic Escherichia coli (ExPEC) lineages. *Clin. Microbiol. Rev.* **32**, 10–1128 (2019). 10.1128/CMR.00135-18 PMC6589867 31189557

[96] Bahgat, O. T., Rizk, D. E., Kenawy, H. I. & Barwa, R. Prevalence of E. coli pathotypes: a comparative study between clinical and environmental isolates. Egypt. *J. Med. Microbiol.* **32**, 59–69 (2023).

[97] Braz, V. S., Melchior, K. & Moreira, C. G. *Escherichia coli* as a multifaceted pathogenic and versatile bacterium. *Front. Cell. Infect. Microbiol.* **10**, 548492 (2020). 33409157 10.3389/fcimb.2020.548492 PMC7779793

[98] Santos, A. C. D. M., Santos, F. F., Silva, R. M. & Gomes, T. A. T. Diversity of hybrid-and hetero-pathogenic Escherichia coli and their potential implication in more severe diseases. *Front. cell. Infect. Microbiol.* **10**, 339 (2020). 32766163 10.3389/fcimb.2020.00339 PMC7381148

[99] Chen, H., Wang, M., Lin, X., Shi, C. & Liu, Z. Bacterial microbiota profile in gills of modified atmosphere-packaged oysters stored at 4°C. *Food Microbiol.* **61**, 58–65 (2017). 27697170 10.1016/j.fm.2016.08.006

[100] Yu, M., Wang, X. & Yan, A. Microbial profiles of retail pacific oysters (Crassostrea gigas) from Guangdong Province, China. *Front. Microbiol.* **12**, 689520 (2021). 34305851 10.3389/fmicb.2021.689520 PMC8292972

