# Supplementary material for: Public health concern of antimicrobial resistance and virulence determinants in E. coli isolates from oysters in Egypt
Source: Sci Rep. 2024 Nov 6;14:26977. doi: 10.1038/s41598-024-77519-y (PMC11541584; doi:10.1038/s41598-024-77519-y)
Supplement: Supplementary file 1 — Supplementary Material 1 [file 41598_2024_77519_MOESM1_ESM.docx]

**S1.** Sequence of oligonucleotide primers used for PCR amplification of β-lactamase and carbapenemase-encoding genes

| **Genes** | **Primer sequence (5ʹ–3ʹ)** | **Amplicon**  **Size (bp)** | **References** |
| --- | --- | --- | --- |
| β lactamase (ESBLs)- encoding genes | | | |
| *bla*_TEM_ | F:CGCCGCATACACTATTCTCAGAATGA  R:ACGCTCACCGGCTCCAGATTTAT | 445 | [54, 55] |
| *bla*_SHV_ | F:CTTTATCGGCCCTCACTCAA  R:AGGTGCTCATCATGGGAAAG | 237 |  |
| *bla*_CTX-M_ | F:ATGTGCAGYACCAGTAARGTKATGGC  R:TGGGTRAARTARGTSACCAGAAYC AGC GG | 593 |  |
| *bla*_OXA-1_ | F:ACA CAA TAC ATA TCA ACT TCG C  R:AGT GTG TTT AGA ATG GTG ATC | 813 |  |
| *bla*_CMY-2_ | F:AGCGATCCGGTCACGAAATA  R:CCCGTTTTATG CACCCATGA | 695 | [56] |
| Carbapenemase- encoding genes | | | |
| *bla*_KPC_ | F:ATG TCA CTG TAT CGC CGT CT  R: TTT TCA GAG CCT TAC TGC CC | 882 | [57] |
| *bla*_NDM_ | F:GGT TTG GCG ATC TGG TTT TC  R:CGG AAT GGC TCA TCA CGA TC | 621 |  |
| *bla*_VIM_ | F:AGT GGT GAG TAT CCG ACAG  R:ATG AAA GTG CGT GGA GAC | 261 | [58] |
| *bla*_OXA-48_ | F :GCTTGATCGCCCTCGATT  R: GATTTGCTCCGTGGCCGAAA | 283 | [59] |

**S2. The list of genes that were selected for virulence gene screening, categorized based on their association with *E. coli* pathotypes.**

| **Pathotypes** | **Virulent**  **genes** | **Primer sequence (5–3)** | **Annealing**  **( ◦C)** | **Amplicon**  **size (bp)** | **References** |
| --- | --- | --- | --- | --- | --- |
| EXPEC | *sfa* | F:CTCCGGAGAACTGGGTGCATCTTAC  R:CGGAGGAGTAATTACAAACCTGGCA | 64 | 410 | [29, 41] |
|  | *cnf1* | F:AAGATGGAGTTTCCTATGCAGGAG  R:CATTCAGAGTCCTGCCCTCATTATT | 63 | 498 |  |
|  | *papC* | F:GTGGCAGTATGAGTAATGACCGTTA  R:ATATCCTTTCTGCAGGGATGCAATA | 64 | 200 |  |
|  | *hlyA* | F:AACAAGGATAAGCACTGTTCTGGC  R:ACCATATAAGCGGTCATTCCCGTCA | 63 | 1177 |  |
|  | *rfc* | F:ATCCATCAGGAGGGGACTGGA  R:AACCATACCAACCAATGCGAG | 63 | 788 |  |
|  | *sepA* | F:TAAAACCCGCCGCCTGAGTA  R:TGCCGGTGAACAGGAGGTTT | 62 | 611 |  |
| EAEC | *etrA* | F:CTTCTTCCTAACGAAACTATCATTA  R:TGACATATCAACTTTCTCTTACGC | 55 | 913 |  |
| EIEC | *aer* | F:TACCGGATTGTCATATGCAGACCGT  R:AATATCTTCCTCCAGTCCGGAGAAG | 60 | 602 |  |
| ETEC | *faeG* | F:GAATCTGTCCGAGAATATCA  R:GTTGGTACAGGTCTTAATGG | 55 | 499 |  |
|  | *fasA* | F:GTAACTCCACCGTTTGTATC  R:AAGTTACTGCCAGTCTATGC | 62 | 409 |  |
|  | *eltA* | F:GGCGTTACTATCCTCTCTAT  R:TGGTCTCGGTCAGATATGT | 55 | 272 |  |
|  | *estA* | F:CAACTGAATCACTTGACTCTT  R:TTAATAACATCCAGCACAGG | 55 | 158 |  |
| EPEC | *eaeA* | F:GACCCGGCACAAGCATAAGC  R:CCACCTGCAGCAACAAGAGG | 63 | 384 |  |
|  | *exhA* | F:GCATCATCAAGCGTACGTTCC  R:AATGAGCCAAGCTGGTTAAGCT | 63 | 534 |  |
| EHEC | *stx1* | F:TGTCGCATAGTGGAACCTCA  R:TGCGCACTGAGAAGAAGAGA | 58 | 655 |  |
|  | *stx2* | F:CCATGACAACGGACAGCAGTT  R:TGTCGCCGATTATCTGACATTC | 58 | 477 |  |

**S3.** Phenotypic and genotypic resistance profile of *E. coli* isolated from retail oysters in Egypt

| ***E .coli* isolates** | **phenotypic pattern** | | | | | | | | | | | | | | | **genotypic pattern** | | | | | | | | | **Resistance profile** | | | |
| --- | --- | --- | --- | --- | --- | --- | --- | --- | --- | --- | --- | --- | --- | --- | --- | --- | --- | --- | --- | --- | --- | --- | --- | --- | --- | --- | --- | --- |
|  | **AMP** | **FOX** | **CTX** | **CRO** | **CAZ** | **MRP** | **ETP** | **AK** | **AT** | **Eo** | **Do** | **CIP** | **LE** | **COT** | **C** | ***bla* _TEM_** | ***bla* _SHV_** | ***bla* _CTX-M_** | ***bla* _OXA.1_** | ***bla* _CMY-2_** | ***bla* _VIM_** | ***bla* _OXA.48_** | ***bla* _NDM_** | ***bla* _KPC_** |  |  |  |  |
|  |  |  |  |  |  |  |  |  |  |  |  |  |  |  |  |  |  |  |  |  |  |  |  |  | **MDR** | **ESBL** | **Plasmid mediated AMPC** | **Carbapenem resistant** |
| E.4 | R | S | R | S | R | R | R | S | S | R | R | R | I | R | I | + | - | + | - | - | - | + | + | - | √ | √ |  | √ |
| E.5 | R | S | R | S | R | I | I | I | S | R | I | I | S | R | I | + | - | - | - | - | - | - | - | + | √ | √ |  | √ |
| E.9 | R | S | R | S | R | R | R | R | S | R | S | I | S | S | R | + | - | - | + | - | - | - | + | + | √ | √ |  | √ |
| E.12 | R | S | R | S | R | R | R | S | S | R | S | S | S | I | S | + | - | - | - | - | - | - | + | + | √ | √ |  | √ |
| E.21 | S | S | R | R | R | R | R | R | S | R | S | S | S | R | R | + | - | - | - | - | - | + | + | + | √ | √ |  | √ |
| E.1 | R | S | R | I | R | R | R | S | S | R | R | R | I | R | R | + | + | + | - | + | - | + | + | - | √ |  | √ | √ |
| E.20 | I | S | S | R | I | R | R | S | S | R | I | I | S | S | S | + | + | - | - | + | - | + | + | + | √ |  | √ | √ |
| E.23 | R | S | R | S | R | R | R | R | S | R | S | S | S | S | R | + | - | - | - | + | + | + | + | + | √ |  | √ | √ |
| E.32 | R | S | R | S | I | R | R | S | R | R | I | I | S | R | S | + | + | - | - | + | + | + | + | + | √ |  | √ | √ |
| E.11 | R | S | R | R | R | S | S | I | S | R | R | R | I | S | S | + | - | + | - | - | - | - | - | - | √ | √ |  |  |
| E.13 | R | S | R | S | R | S | S | R | S | R | R | R | S | R | R | + | - | - | - | - | - | - | - | - | √ | √ |  |  |
| E.15 | R | I | I | I | R | S | S | S | R | R | S | S | S | R | S | + | - | - | - | - | - | - | - | - | √ | √ |  |  |
| E.17 | R | R | R | R | R | S | S | I | S | R | S | S | S | S | S | - | - | - | - | - | - | - | - | - | √ | √ |  |  |
| E.16 | S | S | I | S | I | S | S | I | S | R | S | S | S | R | S | - | - | + | + | + | - | - | - | - | √ |  | √ |  |
| E.18 | I | I | I | I | I | S | S | R | S | R | I | I | S | S | S | + | - | - | - | + | - | - | - | - | √ |  | √ |  |
| E.19 | S | S | S | S | S | S | S | R | S | R | S | S | S | I | S | + | - | - | - | + | - | - | - | - | √ |  | √ |  |
| E.24 | S | S | R | S | I | R | R | S | R | R | S | S | S | S | S | + | - | - | - | + | + | + | + | + |  |  | √ | √ |
| E.25 | S | S | I | S | S | R | R | S | S | R | S | S | S | S | S | + | - | - | - | + | + | - | - | + |  |  | √ | √ |
| E.26 | S | R | I | S | R | R | R | S | S | R | S | S | S | S | S | + | - | - | - | + | + | + | - | + |  |  | √ | √ |
| E.2 | R | S | R | S | R | R | R | S | S | R | S | S | S | S | S | + | + | + | - | - | + | + | + | + |  | √ |  | √ |
| E.14 | R | S | R | S | R | S | S | S | S | R | S | S | S | S | S | + | - | - | - | - | - | - | - | - |  | √ |  |  |
| E.3 | S | S | R | S | I | S | S | S | S | R | S | S | S | S | S | + | - | - | - | + | - | - | - | - |  |  | √ |  |
| E.6 | R | S | I | S | S | S | S | S | S | R | S | S | S | S | S | + | - | - | - | + | - | - | - | - |  |  | √ |  |
| E.7 | I | S | R | I | I | S | S | S | S | R | S | S | S | S | S | + | - | - | - | + | - | - | - | - |  |  | √ |  |
| Total | | | | | | | | | | | | | | | | 22 | 4 | 5 | 2 | 13 | 6 | 9 | 10 | 11 | 16 | 11 | 13 | 12CR, 1CI= 13 |
| % | | | | | | | | | | | | | | | | 91.7 | 16.7 | 20.8 | 8.3 | 54.2 | 25 | 37.5 | 41.7 | 45.8 | 66.7 | 45.8 | 54.2 |  |

Antibiotic discs: Ampicillin (AMP), Cefoxitin (FOX), Cefotaxime (CTX), Ceftriaxone (CRO), Ceftazidime (CAZ), Meropenem (MRP), Ertapenem (ETP), Amikacin (AK), Azithromycin (AT), Erythromycin (Eo), Doxycycline (Do), Ciprofloxacin (CIP), Levofloxacin (LE), Trimethoprim-Sulfamethoxazole (COT), and Chloramphenicol(C).

**S4.** Occurrence of virulence genes categorized based on their association with pathotypes in *E. coli* isolates recovered from retail oysters in Egypt

| ***E. coli* isolates** | **EXPEC** | | | | | | **EPEC** | | **EHEC** | | **ETEC** | | | | **EIEC** | **EAEC** | **Pathotypes** |
| --- | --- | --- | --- | --- | --- | --- | --- | --- | --- | --- | --- | --- | --- | --- | --- | --- | --- |
|  | ***sfa*** | ***cnf1*** | ***papC*** | ***hlyA*** | ***rfc*** | ***sepA*** | ***eaeA*** | ***exhA*** | ***stx1*** | ***stx2*** | ***faeG*** | ***fasA*** | ***eltA*** | ***estA*** | ***aer*** | ***etrA*** |  |
| E.1 | + | - | + | - | - | - | + | + | - | + | - | - | - | + | + | - | EIEC,EHEC,EPEC,ETEC,EXPEC |
| E.2 | + | - | + | - | - | - | - | - | - | - | - | - | - | - | - | - | EXPEC |
| E.3 | - | - | + | - | - | - | + | - | - | + | - | - | - | - | - | - | EHEC,EPEC,EXPEC |
| E.4 | + | - | + | - | - | - | + | - | + | + | - | - | - | - | + | - | EIEC,EHEC,EPEC,EXPEC |
| E.5 | + | - | + | - | - | - | - | + | + | + | - | - | - | + | + | - | EIEC,EHEC,ETEC,EXPEC,EPEC |
| E.6 | + | - | + | - | - | - | + | + | + | - | - | - | - | + | - | - | EHEC,EPEC, ETEC,EXPEC |
| E.7 | - | - | - | - | - | - | - | + | - | - | - | - | - | - | - | - | EPEC |
| E.9 | + | - | + | + | + | - | + | + | + | - | - | - | - | + | - | - | EXPEC,EHEC,EPEC,ETEC |
| E.11 | - | - | + | - | - | - | - | - | - | - | - | - | - | - | - | - | EXPEC |
| E.12 | + | - | + | + | + | - | + | - | + | + | - | - | - | + | + | - | EIEC,EHEC,EXPEC,EPEC,ETEC |
| E.13 | + | - | + | - | - | - | - | + | - | - | - | - | - | - | - | - | EXPEC,EPEC |
| E.14 | + | - | + | - | + | - | + | + | - | - | - | - | - | + | - | - | EXPEC, EPEC, ETEC |
| E.15 | - | - | + | - | - | - | - | + | - | - | - | - | - | - | - | - | EXPEC, EPEC |
| E.16 | + | - | + | - | - | - | + | + | - | - | - | - | - | + | - | - | EPEC,ETEC,EXPEC |
| E.17 | - | - | - | - | - | - | - | - | - | - | - | - | - | - | - | - | Negative |
| E.18 | + | - | + | - | - | - | - | + | - | - | - | - | - | + | - | - | ETEC,EXPEC, EPEC |
| E.19 | + | - | + | + | - | - | - | - | - | + | - | - | - | - | - | - | EHEC,EXPEC |
| E.20 | - | - | + | + | - | - | - | + | - | - | - | - | - | - | + | - | EIPEC,EXPEC,EPEC |
| E.21 | + | - | + | - | + | - | + | + | + | - | - | - | - | - | + | - | EIEC,EHEC,EXPEC,EPEC |
| E.23 | + | - | + | - | + | - | + | - | + | + | - | - | - | - | + | + | EIEC,EHEC,EAEC,EXPEC,EPEC |
| E.24 | - | + | + | - | - | - | - | - | - | + | - | - | - | + | + | - | EIEC,EHEC,EXPEC,ETEC |
| E.25 | + | - | + | - | + | - | - | - | + | - | - | - | - | + | + | + | EIEC,EAEC,EHEC,EXPEC,ETEC |
| E.26 | - | - | - | - | - | - | - | - | - | - | - | - | - | - | - | - | Negative |
| E.32 | + | - | + | + | - | - | + | - | + | - | - | - | - | + | + | + | EIEC,EAEC,EXPEC,EHEC,EPEC,ETEC |
| Total | 16 | 1 | 21 | 5 | 6 | 0 | 11 | 12 | 9 | 8 | 0 | 0 | 0 | 11 | 10 | 3 |  |
| % | 66.7 | 4.2 | 87.5 | 20.8 | 25 | 0 | 45.8 | 50 | 37.5 | 33.3 | 0 | 0 | 0 | 45.8 | 41.7 | 12.5 |  |

**S5.** Distribution of virulence genes and their association with antimicrobial resistance among MDR *Escherichia coli* (n=16) isolates from oysters in Egypt

| **MDR**  ***E. coli* isolates** | **Antimicrobial resistance pattern** | | | | | | | | | | | | | | | **Virulence genes** | | | | | | | | | | | | | | | |
| --- | --- | --- | --- | --- | --- | --- | --- | --- | --- | --- | --- | --- | --- | --- | --- | --- | --- | --- | --- | --- | --- | --- | --- | --- | --- | --- | --- | --- | --- | --- | --- |
|  | **AMP** | **FOX** | **CTX** | **CRO** | **CAZ** | **MRP** | **ETP** | **AK** | **AT** | **Eo** | **Do** | **CIP** | **LE** | **COT** | **C** | ***sfa*** | ***cnf1*** | ***papC*** | ***hlyA*** | ***rfc*** | ***sepA*** | ***eaeA*** | ***exhA*** | ***stx1*** | ***stx2*** | ***faeG*** | ***fasA*** | ***eltA*** | ***estA*** | ***aer*** | ***etrA*** |
| E.1 | R | S | R | I | R | R | R | S | S | R | R | R | I | R | R | + | - | + | - | - | - | + | + | - | + | - | - | - | + | + | - |
| E.4 | R | S | R | S | R | R | R | S | S | R | R | R | I | R | I | + | - | + | - | - | - | + | - | + | + | - | - | - | - | + | - |
| E.5 | R | S | R | S | R | I | I | I | S | R | I | I | S | R | I | + | - | + | - | - | - | - | + | + | + | - | - | - | + | + | - |
| E.9 | R | S | R | S | R | R | R | R | S | R | S | I | S | S | R | + | - | + | + | + | - | + | + | + | - | - | - | - | + | - | - |
| E.11 | R | S | R | R | R | S | S | I | S | R | R | R | I | S | S | - | - | + | - | - | - | - | - | - | - | - | - | - | - | - | - |
| E.12 | R | S | R | S | R | R | R | S | S | R | S | S | S | I | S | + | - | + | + | + | - | + | - | + | + | - | - | - | + | + | - |
| E.13 | R | S | R | S | R | S | S | R | S | R | R | R | S | R | R | + | - | + | - | - | - | - | + | - | - | - | - | - | - | - | - |
| E.15 | R | I | I | I | R | S | S | S | R | R | S | S | S | R | S | - | - | + | - | - | - | - | + | - | - | - | - | - | - | - | - |
| E.16 | S | S | I | S | I | S | S | I | S | R | S | S | S | R | S | + | - | + | - | - | - | + | + | - | - | - | - | - | + | - | - |
| E.17 | R | R | R | R | R | S | S | I | S | R | S | S | S | S | S | - | - | - | - | - | - | - | - | - | - | - | - | - | - | - | - |
| E.18 | I | I | I | I | I | S | S | R | S | R | I | I | S | S | S | + | - | + | - | - | - | - | + | - | - | - | - | - | + | - | - |
| E.19 | S | S | S | S | S | S | S | R | S | R | S | S | S | I | S | + | - | + | + | - | - | - | - | - | + | - | - | - | - | - | - |
| E.20 | I | S | S | R | I | R | R | S | S | R | I | I | S | S | S | - | - | + | + | - | - | - | + | - | - | - | - | - | - | + | - |
| E.21 | S | S | R | R | R | R | R | R | S | R | S | S | S | R | R | + | - | + | - | + | - | + | + | + | - | - | - | - | - | + | - |
| E.23 | R | S | R | S | R | R | R | R | S | R | S | S | S | S | R | + | - | + | - | + | - | + | - | + | + | - | - | - | - | + | + |
| E.32 | R | S | R | S | I | R | R | S | R | R | I | I | S | R | S | + | - | + | + | - | - | + | - | + | - | - | - | - | + | + | + |
| Total | | | | | | | | | | | | | | | | 12 | 0 | 15 | 5 | 4 | 0 | 8 | 9 | 7 | 6 | 0 | 0 | 0 | 7 | 8 | 2 |
| % | | | | | | | | | | | | | | | | 75 | 0 | 93.8 | 31.3 | 25 | 0 | 50 | 56.3 | 43.8 | 37.5 | 0 | 0 | 0 | 43.8 | 50 | 12.5 |

Antibiotic discs: Ampicillin (AMP), Cefoxitin (FOX), Cefotaxime (CTX), Ceftriaxone (CRO), Ceftazidime (CAZ), Meropenem (MRP), Ertapenem (ETP), Amikacin (AK), Azithromycin (AT), Erythromycin (Eo), Doxycycline (Do), Ciprofloxacin (CIP), Levofloxacin (LE), Trimethoprim-Sulfamethoxazole (COT), and Chloramphenicol(C).
